# A cross-sectional observational study about media and infection control practices: are photographic portrayals of healthcare workers setting a bad example?

**DOI:** 10.1186/s13756-015-0094-z

**Published:** 2015-11-25

**Authors:** E. J. W. Spierings, P. T. J. Spierings, M. Nabuurs-Franssen, J. Hopman, E. Perencevich, A. Voss

**Affiliations:** Radboud University of Nijmegen, Medical School, Platolaan 340, 6525 KD Nijmegen, The Netherlands; Spierings Medische Techniek B.V., Nijmegen, The Netherlands; Department of Medical Microbiology and Infectious Diseases, Canisius-Wilhelmina Hospital, Nijmegen, The Netherlands; Department of Medical Microbiology, Radboud University of Nijmegen, Nijmegen, The Netherlands; Division of Infectious Diseases and Epidemiology, University of Iowa Hospital and Clinics, Iowa City, IA USA

## Abstract

**Background:**

Attempts to increase compliance with infection control practices are complex and are - in part - based on attempts to change behaviour. In particular, the behaviour of significant peers (role models) has been shown to be a strong motivator. While role models within the working environment are obviously the most important, some experts suggest that media and public display cannot be ignored. The aim of this present study was to examine the display of technique recommended by current infection control guidelines including the “bare below the elbow” principle, which is considered a basic requirement for good infection control in many countries, in sets of professional stock photos.

**Findings:**

From 20 random photo-stock websites we selected pictures with search terms “doctor and patient” and “nurse and patient”. In all selected photos a doctor or nurse and a patient were presented, healthcare workers (HCWs) were wearing white coats or uniforms, and their arms were visible. Each photo was evaluated with regard to: closure of white coat, sleeve length, personal clothing covered, hairstyle and presence of a wristwatch, bracelet and/or ring. Overall, 1600 photos were evaluated.

The most common mistakes were with regard to HCWs’ white coats/uniforms. Eighty-nine percent of the photos containing doctor’s images were considered incorrect while 28 % of nurse-containing photos were incorrect.

**Conclusions:**

The results seem to reflect the real world with only 40 % displaying correct behaviour with doctors being worse than nurses. It seems that the stereotypical image of a doctor does not agree with the current infection control guidelines. If we aim for higher compliance rates of HCWs, we need to change the social image of doctors and improve production, selection and display of stock photo images.

## Introduction

One of the greatest challenges facing modern healthcare are nosocomial infections. They affect almost 10 % of hospitalized patients, and are responsible for prolonged hospital stays, substantial morbidity and mortality and excess costs [1]. Furthermore, multidrug-resistant pathogens are often involved in healthcare-associated infections and impede effective treatment. Healthcare-associated pathogens are commonly transmitted via the hands of healthcare workers (HCWs) from patient to patient and within the healthcare environment [2–5]. To prevent antimicrobial resistant pathogens from spreading and to reduce healthcare-associated infections (HAIs), optimal hand hygiene is essential [3].

The risk of hand contamination and the effect of hand hygiene are influenced by many factors. HCWs wearing wristwatches or rings is an important factor [6]. Several studies identified an association between ring wearing and an enhanced bacterial load on hands [7–9], whereas others show the same effect as a consequence of wearing wristwatches [10, 11]. These studies yield the conclusion that watch wearers have higher counts of bacteria on their wrist compared to HCW’s without a wristwatch. In addition, it has been demonstrated that the white coats of doctors, especially the long sleeves, are often bacteriologically contaminated [12] as well as that those doctors wearing long sleeves are more likely to miss areas of the wrist during washing [13].

The main objective of the First Global Patient Safety Challenge, launched by the World Health Organization (WHO), is to achieve a strong patient safety culture by improving hand hygiene practices worldwide [4]. Improvement in adherence to recommended hand hygiene guidelines is necessary to achieve this goal. Compliance with hand hygiene guidelines in healthcare institutions remains unacceptably low, and until recently rarely exceeded 40 % [14]. Attempts to increase compliance are frequently met with little success [15]. It appears that true behavioural change cannot be achieved by targeting the individual alone. The organisational environment surrounding the individual HCW must also be adressed [16], which makes promotion of hand hygiene behaviour a complex issue.

Ponce de Leon et al. suggested that what we see in the media (in their study the TV-show ER) could influence the behaviour of HCWs [17]. In general, the educational effect of the media cannot be ignored. In our own institution, as well as in other national and international publications, we frequently utilize stock photos, which could convey correct or misleading impressions of appropriate behaviour to HCWs. Additionally, many medical magazines use stock photo websites as supplier for images. These photos are not always realistic and often ignore current infection control guidelines, thereby spreading “mixed-messages” to HCWs, and consequently may negatively influence their behaviour. The aim of the present study was to examine the display of technique recommended by current infection control guidelines including the “bare below the elbow” principle, which is considered a basic requirement for good hand hygiene in many countries, in sets of professional stock databases.

## Methods

Searches for eligible stock websites were conducted using Google. We selected at random a convenience sample of 20 large photo stock websites with a minimum of five million pictures: 123rf, photos, fotosearch, inmagine, stocklib, fotolia, gettyimages, istockphoto, jupiterimages, dreamstime, thinkstock, shutterstock, bigstockphoto, canstockphoto, photoxpress, imageselect, reflexstock, depositphotos, stockfresh and veer. From each website we selected pictures with the search term “doctor and patient” and pictures with the search term “nurse and patient”. It is not possible to determine absolutely wether the HCW shown in the picture is a doctor or a nurse. We relied on how the stock photos were tagged. The first 40 pictures for each search term that met the inclusion criteria were selected. A total of 1600 photos were selected for review. Photos were included if they met the following criteria; a doctor or nurse is present, a patient is present, the doctor or nurse wears work clothes, and both arms of the doctor or nurse are visible. Photos were analysed according to the seven criteria in Table 1 and classified as correct or incorrect. If an error is not shown then it is determined correct.

**Table 1:** The seven criteria used to analyse the photos and classify them as correct or incorrect

Criteria	Correct	Incorrect
Uniform	Closed uniform or one top button open	Open or half open uniform
Sleeve length	Elbow visible	Elbow not visible
Covering of personal sleeves	Personal sleeves covered	Personal sleeves visible
Watch	Absent	Visible
Bracelet	Absent	Visible
Ring	Absent	Visible
Hairstyle	Short or tied	Long and loose, able to contact patient

**Table 2:** Percentage of incorrect photos by type of HCW per website

Website	Incorrect photos of doctors (%)	Incorrect photos of nurses (%)
www.123rf.com	82.5	27.5
www.photos.com	87.5	17.5
www.fotosearch.com	87.5	17.5
www.inmagine.com	90.0	32.5
www.stocklib.com	92.5	17.5
www.fotolia.com	95.0	22.5
www.gettyimages.nl	92.5	32.5
www.istockphoto.com	100.0	35.0
www.jupiterimages.com	95.0	30.0
www.dreamstime.com	95.0	22.5
www.thinkstock.com	90.0	20.0
www.shutterstock.com	87.5	12.5
www.bigstockphoto.com	87.5	32.5
www.canstockphoto.com	87.5	22.5
www.photoxpress.com	77.5	50.0
www.imageselect.eu	82.5	45.0
www.reflexstock.com	85.0	35.0
www.depositphotos.com	85.0	32.5
www.stockfresh.com	82.5	25.0
www.veer.com	92.5	20.0

Polished nails and length of nails were not reviewed, due to the fact that an adequate review of nails was not possible on most photos.

The criteria in Table 1 are in accordance with the protocol “Personal hygiene of health care workers” of the Dutch Working Party for Infection Control (WIP) [18]. The WIP infection control guidelines are seen as a national standard and those dealing with hand hygiene are based on the guidelines of the WHO.

The total amount of incorrect photos was determined for both groups of healthcare workers. The difference between both groups and their corresponding 95 % confidence interval was calculated. The number of mistakes found per criteria were determined and compared between both groups.

## Results

**Fig. 1:**
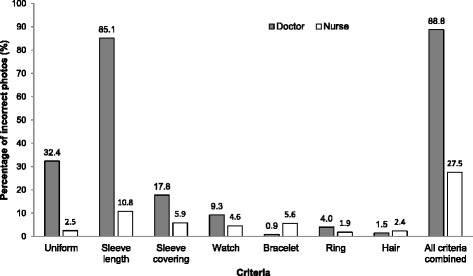
Percentage of incorrect photos per criterion and type of healthcare-worker

In total, 800 photos with doctors and 800 with nurses were selected. Based on the selected criteria (Table 1) 710 (88.8 %) from the 800 photos with a doctor present contained at least one incorrect behaviour and 220 (27.5 %) from 800 photos with a nurse present were incorrect in at least on element. The difference in incorrectly classified photos between both groups was 61.3 % (CI 57.5 % – 65.0 %). The number of incorrect photos per website ranged from 77.5 % – 100 % for those showing doctors, and from 12.5 % - 50 % for those displaying nurses (Table 2). Figure 1 summarizes the main results, by showing the percentage of incorrect behaviour per criterion stratified by type of HCW.

## Discussion

In a large representative sample of stock photos available on the internet, a large proportion of doctors images contained at least one incorrect behaviour. Doctor’s images were three times more likely to contain incorrect behaviour compared to nurses. This discrepancy was primarily driven by the doctors wearing uniforms with long sleeves with their uniform not properly closed. The image of a doctor according to photo-stock pictures was typically a male with a long-sleeved white coat. Only 11 % of the photos in which a doctor is presented can be rated as entirely correct. This in contrast to the photos in which a nurse is presented of which 69 % were rated as correct. In the images, doctors were more likely to be displayed wearing watches and rings than nurses. The only criteria that was more frequently wrong in pictures with nurses were bracelet and hairstyle. Obviously, an old-fashioned gender bias of stock-photo makers is a confounding factor in the comparison between doctors and nurses. During the study we observed that almost all nurses were female, consequently increasing the chance of wrongly wearing bracelets and longer or loose hair.

## Conclusion

**Fig. 2:**
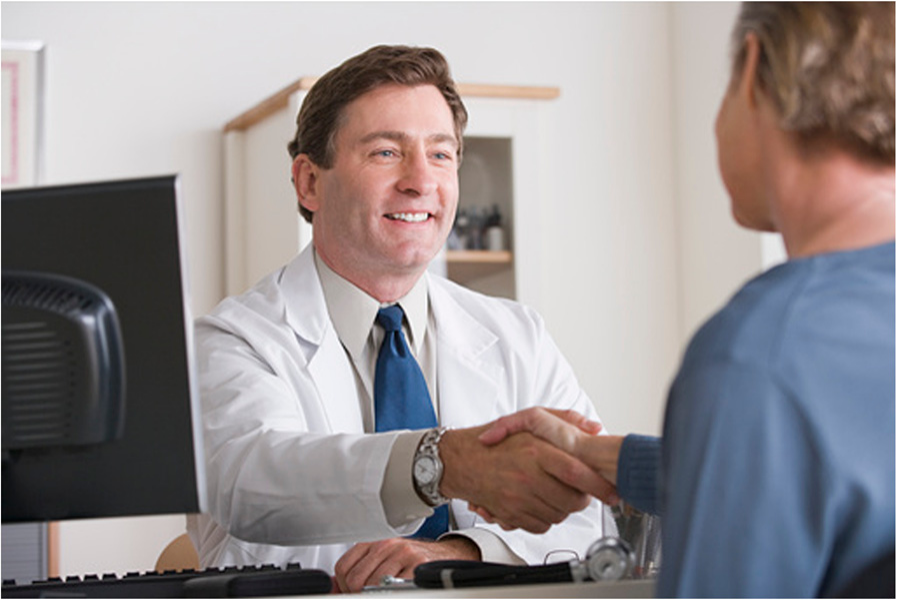
Example of a faulty photo. Doctor with open uniform, long sleeves, personal sleeve visible and wearing a watch

**Fig. 3:**
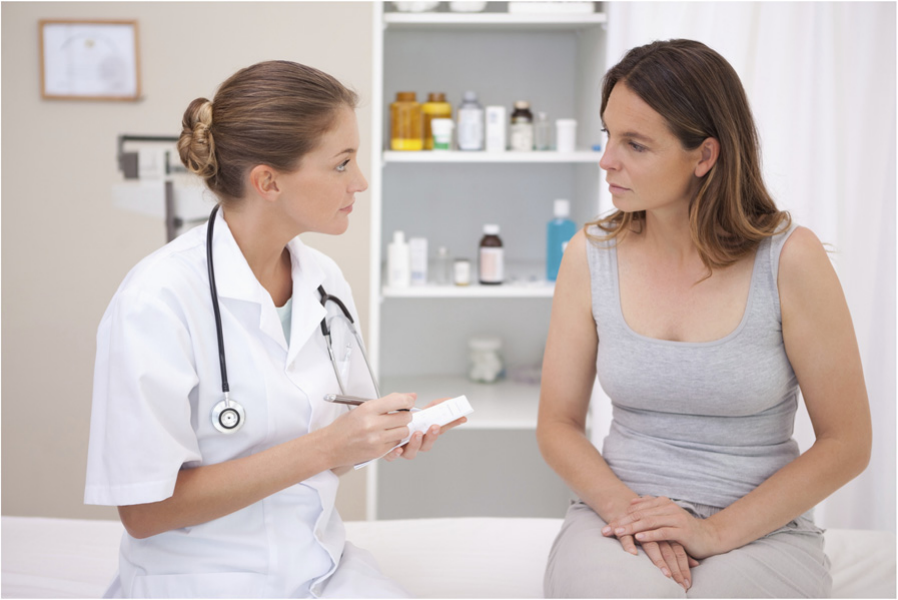
Example of a correct photo. Doctor with correct hairstyle, closed uniform, short sleeves and not wearing a watch, ring or bracelet

The results show that infection control guidelines are largely ignored by currently available stock photos. Appropriate clothing of doctors in these photos were extremely rare. The overall image of a doctor in the stock photos is a handsome, middle-aged male with an open white coat, his tie is explicitly visible and he is wearing a watch, as shown in Fig. 2. An example of a correct photo is shown in Fig. 3. It seems that the stereotype image of a doctor does not agree with the current infection control guidelines. If we aim for higher compliance rates of HCWs, we need to change the social image of doctors. The media should be aware that they have a responsibility as well. It should be pointed out that HCWs might be affected by pictures showing a faulty example. Until stock photos are updated to reflect current best-practice for infection control, they should not be utilized in hospital or other healthcare settings. Until that time, we recommend that facilities use only in-house images of clinicians displaying proper attire and behaviour.
